# Bidirectional Relationship Between Subjective Age and Frailty: Findings From the NHATS

**DOI:** 10.1093/geroni/igaa057.867

**Published:** 2020-12-16

**Authors:** Yuxiao Li, Minhui Liu, Christina Miyawaki, Xiaocao Sun, Tianxue Hou, Siyuan Tang, Sarah Szanton

**Affiliations:** 1 Central South University, Changsha, Hunan, China; 2 University of Houston, Houston, Texas, United States; 3 Johns Hopkins University, Baltimore, Maryland, United States

## Abstract

Frailty is a clinical syndrome that becomes increasingly common as people age. Subjective age refers to how young or old individuals experience themselves to be. It is associated with many risk factors of frailty, such as increased depression, worse cognitive function, and poorer psychological wellbeing. In this study, we examined the relationship between subjective age and frailty using the 2011-2015 waves of the National Health and Aging Trends Study. Participants were community-dwelling older adults without frailty in the initial wave (N=1,165). Subjective age was measured by asking participants, “What age do you feel most of the time?” Based on the Fried five phenotypic criteria: exhaustion, unintentional weight loss, low physical activity, slow gait, and weak grip strength, frailty was categorized into robust=0, pre-frail=1 or 2; frail=3 or more criteria met. Participants were, on average, 74.1±6.5 years old, female (52%), and non-Hispanic White (81%). Eighty-five percent of the participants felt younger, and 3% felt older than their chronological age, but 41% of them were pre-frail/frail. Generalized estimating equations revealed that an “older” subjective age predicted a higher likelihood of pre-frailty and frailty (OR, 95%CI= 1.01, 1.01-1.02). In contrast, frailty predicted an “older” subjective age (OR, 95%CI= 2.97, 1.65-5.35) adjusting for demographics and health conditions. These findings suggest a bidirectional relationship between subjective age and frailty. Older people who feel younger than their chronological age are at reduced risk of becoming pre-frail/frail. Intervention programs to delay frailty progression should include strategies that may help older adults perceive a younger subjective age.

